# Symptoms associated with influenza vaccination and experimental human pneumococcal colonisation of the nasopharynx

**DOI:** 10.1016/j.vaccine.2020.01.070

**Published:** 2020-02-28

**Authors:** Caz Hales, Simon P. Jochems, Rachel Robinson, Carla Solórzano, Beatriz Carniel, Sherin Pojar, Jesús Reiné, Esther L. German, Elissavet Nikolaou, Elena Mitsi, Angela D. Hyder-Wright, Helen Hill, Hugh Adler, Victoria Connor, Seher Zaidi, Catherine Lowe, Xiaojing Fan, Duolao Wang, Stephen B. Gordon, Jamie Rylance, Daniela M. Ferreira

**Affiliations:** a Royal Liverpool and Broadgreen University Hospital Trust, Prescot Street, Liverpool L7 8XP, UK; b Department of Clinical Sciences, Liverpool School of Tropical Medicine, Pembroke Place, Liverpool L3 5QA, UK

**Keywords:** Controlled human infection challenge model, Influenza vaccination, Pneumococcal inoculation, Live attenuated influenza vaccine, Pneumococcus colonisation, *Streptococcus pneumoniae*, Symptoms

## Abstract

**Background:**

Nasopharyngeal colonisation by *S. pneumoniae* is a prerequisite for invasive pneumococcal infections. Influenza co-infection leads to increased susceptibility to secondary pneumonia and mortality during influenza epidemics. Increased bacterial load and impaired immune responses to pneumococcus caused by influenza play a role in this increased susceptibility. Using an Experimental Human Challenge Model and influenza vaccines, we examined symptoms experienced by healthy adults during nasal co-infection with *S. pneumoniae* and live attenuated influenza virus.

**Methods:**

Randomised, blinded administration of Live Attenuated Influenza Vaccine (LAIV) or Tetravalent Inactivated Influenza Vaccine (TIV) either preceded bacterial inoculation or followed it, separated by a 3-day interval. The presence and density of *S. pneumoniae* was determined from nasal washes. Participants completed a symptom questionnaire from the first intervention until 6 days post second intervention.

**Results:**

The timing and type of influenza vaccination and presence of *S. pneumoniae* in the nasopharynx significantly affected symptom reporting. In the study *where* influenza vaccination *preceded bacterial inoculation*: nasal symptoms were less common in the LAIV group than the TIV group (OR 0.57, p < 0.01); with colonisation status only affecting the TIV group where more symptoms were reported by colonised participants compared to non-colonised participants following inoculation (n = 12/23 [52.17%] vs n = 13/38 [34.21%], respectively; p < 0.05). In the study *where* influenza vaccination *followed bacterial inoculation*: no difference was seen in the symptoms reported between the LAIV and TIV groups following inoculation and subsequent vaccination; and symptoms were unaffected by colonisation status.

**Conclusion:**

Symptoms experienced during live viral vaccination and bacterial co-infection in the nasopharynx are directly affected by the precedence of the pathogen acquisition. Symptoms were directly affected by nasal pneumococcal colonisation but only when TIV was given prior to bacterial exposure.

## Highlights

• The timing and route of influenza vaccines effect symptom reporting in healthy adults.
• Symptoms experienced by healthy adults were further affected by the presence of *S. pneumoniae.*
• LAIV vaccination prior to *S. pneumoniae* exposure/colonisation led to reduced symptoms.
• TIV vaccination prior to *S. pneumoniae* exposure/colonisation led to increased nasal symptoms compared to LAIV.

## 1 Introduction

Nasopharyngeal colonisation by *S. pneumoniae* is a prerequisite for pneumonia, meningitis, sinusitis and otitis media [1], [2]. Reports suggest that point prevalence of colonisation is 21–52% and 6.5–8% in children and adults, respectively [3], [4], [5], although it is uncertain if these natural colonisation events are associated with symptoms [4]. It has been suggested that nasopharyngeal symptoms are related to the viral co-infection and not bacterial colonisation [6]. In children, pneumococcal colonisation densities are increased by viral infection, irrespective of whether symptoms are present or not [6]. Furthermore, Live Attenuated Influenza Vaccine (LAIV) (used annually in children in the UK, USA and other countries) is commonly associated with runny nose and nasal congestion in all ages groups, fever in children, and sore throat in adults [7]. In adults, nasopharyngeal colonisation with *S. pneumoniae* is considered an asymptomatic event [8], [9], [10], [11], although no studies have examined symptoms in the context of viral/bacterial co-infection particularly in adults.

Influenza co-infection can lead to secondary pneumonia, which frequently drives mortality during influenza epidemics [12], [13], [14]. LAIV is safe and effective [15], [16], but it has been reported that it can also increase rates and density of pneumococcal colonisation [17], [18] leading to a theoretical risk of increased pneumococcal transmission to susceptible groups [19].

Using Experimental Human Pneumococcal Challenge (EHPC), we have previously described the interaction of pneumococcus and viral pathogens and host immune responses [18], [20], [21]. Using this model, we now investigated the symptoms experienced by healthy adults during nasal co-infection with influenza and *S. pneumoniae*, and examine the importance of order in which both pathogens are encountered.

## 2 Materials and methods

### 2.1 Trial design and vaccination

This single-centred, double-blinded, randomised, controlled trial involved two sequentially recruited cohorts. The ‘antecedent’ cohort (2015/16) received influenza vaccine 3 days prior to nasal inoculation with *Streptococcus pneumoniae.* The ‘concurrent’ study (2016/17) reversed this order (pneumococcal inoculation was performed 3 days before influenza vaccine administration). Full cohort details have been previously described [18]. The study was EudraCT registered (2014-004634-26).

### 2.2 Sampling, recruitment

We recruited healthy, non-smoking adults, aged 18–50 years old. Exclusion criteria were: influenza or pneumococcal vaccination; clinically confirmed pneumococcal disease in the preceding two years; close contact with ‘high-risk’ individuals (children under 5 years, immunosuppressed, elderly); current febrile illness; recent or current use of antibiotics or immune-modulating medication; pregnancy.

### 2.3 Procedure

The study was conducted as previously described [18]. Briefly, participants were randomised to receive either nasal LAIV (Fluenz Tetra or FluMist Tetra, AstraZeneca, UK; used interchangeably due to procurement shortages; both are pharmaceutically identical but have different packaging and labelling) with an intramuscular placebo (0.5 mL normal saline), or a nasal placebo (0.2 mL normal saline) with an intramuscular Tetravalent Inactivated Influenza Vaccination (TIV) (Fluarix Tetra, GlaxoSmithKline, UK). Participants were blindfolded during administration, with blinding maintained until completion of primary analysis. Experimental pneumococcal challenge comprised 80,000 colony forming-units (CFU) of penicillin-susceptible, mid-log phase grown *Streptococcus pneumoniae* serotype 6B (strain BHN418) in 0.1 mL saline instilled by laboratory pipette into each nostril [22].

### 2.4 Data collection

Pneumococcal colonisation status was determined by conventional culture and quantified by serial dilution of nasal washes [22], [23], [24], [25]. Participants completed a daily symptom questionnaire from the day of vaccination until 6 days post inoculation (antecedent cohort, total of 9 days) or from the day of inoculation until 3 days post vaccination (concurrent cohort, total of 6 days). Participants were unaware of their colonisation status until after symptom log data had been completed and returned. Symptoms were assessed using a modified Likert score for severity [26]. In the antecedent cohort 10 symptoms were examined (5 nasal and 5 non-nasal symptoms) on an 7-point severity scale. Based on participant feedback, the questionnaire was modified for the concurrent cohort to improve usability. The scoring system for both studies was aligned for analysis. A score of ≤2 (no symptoms or occasional limited episode) was considered ‘asymptomatic’ and a score of ≥3 (mild steady symptoms to severe hard to tolerate symptoms) was considered ‘symptomatic’. Symptoms were categorised as indicated to reduce the impact of participants reporting a symptom which was an isolated episode of short duration that would not fit with known symptomology patterns of respiratory infection [27].

### 2.5 Data analysis

Individuals were ‘colonised’ if *S. pneumoniae* serotype 6B was detected by nasal wash culture at any time point following experimental challenge in the study period. Individuals who were naturally colonised with any serotype of *S. pneumoniae* at baseline were excluded from the data analysis. Generalised Estimating Equation (GEE) was used to analyse symptoms with binomial distribution for symptom outcomes (0 = No symptoms, 1 = symptomatic) and logit link function, taking into account repeated measurements in symptoms. To assess the treatment effect on symptoms, the GEE model has treatment (LAIV, TIV), time (<=3 days, >=4 days), interaction between treatment and time as study variables, participants’ age and gender as covariates. Difference in log odds of having a symptomatic outcome between LAIV and TIV at each time point was derived from the GEE model. To assess the difference in symptoms between colonisation status, the GEE model has colonisation status (positive, negative), time (<=3 days, >=4 days), interaction between colonisation status and time as study variables, participants’ age and gender as covariates. Difference in log odds of having a symptomatic outcome between positive and negative colonisation status at each time point was derived from the GEE model. All analysis was based on the intention to treat basis. Statistical analyses were performed using SAS 9.4 (SAS Institute, Cary, NC, USA); a two-tailed P value < 0.05 was considered statistically significant.

### 2.6 Safety and ethics

Adverse events (including symptoms judged to be severe by the clinician), and use of standby antibiotics were universally recorded. No serious adverse events were reported however, 18 participants (antecendent study n = 8; concurrent study n = 10) were reviewed by a clinician for symptoms requiring clinical investigation at any time point in the study. Of the 18 triggered clinical examinations, 6 occurred during the timeframe of daily symptom reporting (see Suppl Table 1). The study was approved by the Liverpool East NHS Research Committee (14-NW-1460) and the Medicines and Healthcare products Regulatory Agency (EudraCT; Protocol 2014-00446334-26). All participants gave informed written consent.

## 3 Main results

### 3.1 Antecedent study

#### 3.1.1 Nasal and non-nasal symptoms increased following TIV vaccination compared to LAIV vaccination

**Fig. 1:**
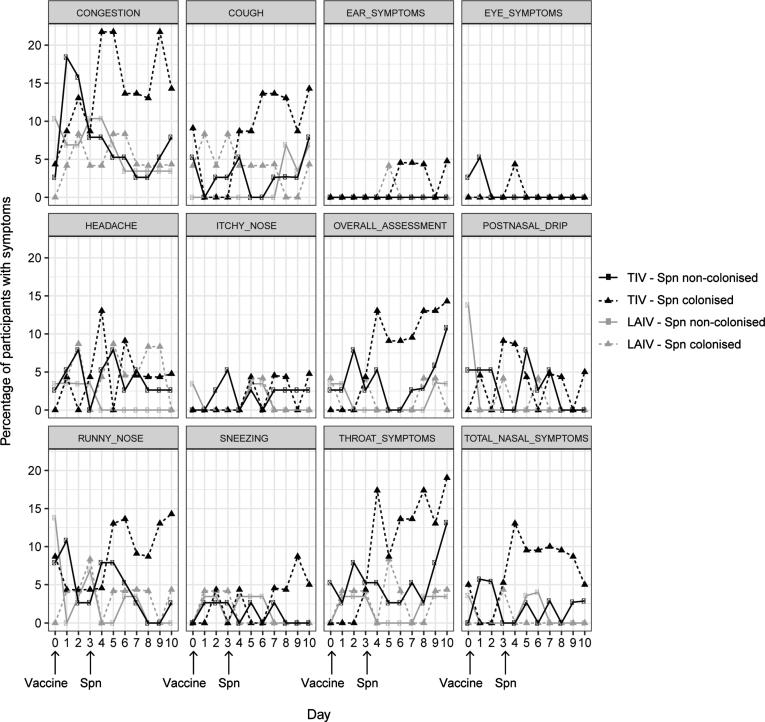
Symptom reporting in the antecedent cohort. The percentage of participants reporting symptoms per day for each of the collected symptoms per group: TIV – Spn non-colonised (n = 38, black circles and solid line), TIV – Spn colonised (n = 23, black triangles and dashed line), LAIV – Spn non-colonised (n = 29, grey circles and solid line), LAIV – Spn colonised (n = 24, grey triangles and dashed line). Day of vaccination and Spn inoculation are depicted. Spn = *S. pneumoniae.*

**Fig. 2:**
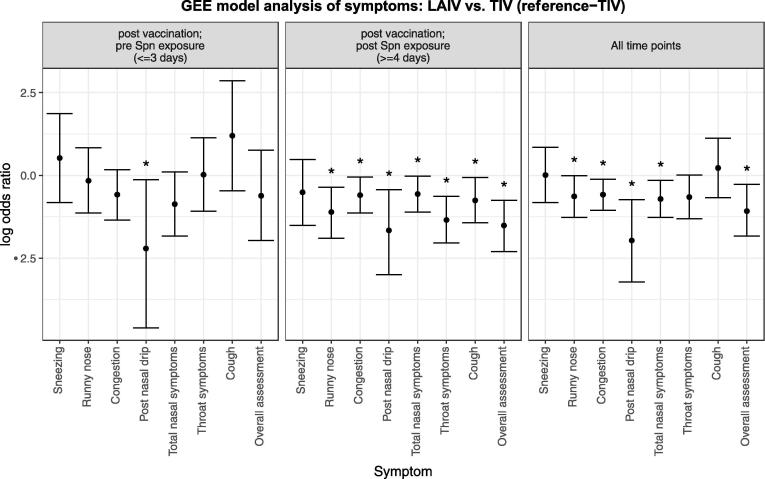
Antecedent study- Symptoms reported by participants receiving nasal LAIV (n = 53) compared with those receiving intramuscular TIV (n = 61). Timepoints are split up as until day of Spn inoculation, post Spn inoculation or all timepoints combined. All subjects are included regardless of ultimate Spn colonisation status. Bars represent log_10_ transformed odds ratios with 95% confidence intervals. Asterisks denote p < 0.05 i.e. a statistically significant difference in the rate of reporting between the two arms of the trial. Odds ratios are derived from GEE model analysis (see methods), and are presented as those occurring early (left panel, at or before 3 days after vaccination and pre Spn inoculation), or late (centre panel, 4 or more days after vaccination and Spn inoculation), or combined throughout the period of reporting (right panel). Spn = *S. pneumoniae.*

Participants were first vaccinated with influenza, and inoculated with *S. pneumoniae* 3 days later. 117 participants completed the primary study end points and 114 returned their symptom log questionnaires for data analysis (LAIV n = 53, TIV n = 61). As previously described, demographics were similar in both groups [18]. 47 participants (41%) became experimentally colonised; 45.3% (n = 24) and 37.7% (n = 23) in the LAIV and TIV groups, respectively. Overall 34.0% (18/53) participants in the LAIV group and 49.2% (30/61) participants in the TIV group reported one or more nasal or non-nasal symptoms, or both, at any time point in the 9 days following vaccination. Statistical difference between these two groups was seen for most nasal symptoms with less symptoms reported in the LAIV group (Fig. 1; Fig. 2 right panel; Suppl Table 2).

#### 3.1.2 Nasal and non-nasal symptoms following TIV vaccination are observed after challenge with *S. pneumoniae*

We investigated whether influenza vaccination led to symptoms in healthy adults. Apart from post nasal drip there was no statistical difference in the reporting of symptoms (LAIV 23%; n = 12/53 vs TIV 25%; n = 15/61) in the 3 days following vaccination in either group (Fig. 1; Fig. 2 left panel; Suppl Table 2). Following inoculation, reporting of one or more symptoms were less in the LAIV group with 19% (n = 10/53) compared to 41% (n = 25/61) in the TIV group. Nasal symptoms were overall less common in the LAIV group (OR 0.57, p < 0.05). Specifically, symptoms of runny nose, congestion and post nasal drip were all significantly less common in the LAIV group (OR 0.33; 0.55; 0.19, respectively; p < 0.05) (Fig. 1; Fig. 2 centre panel; Suppl Table 2). Non-nasal symptoms were also reported less in the LAIV group following inoculation; specifically overall assessment, cough and sore throat (OR 0.22, OR 0.47 and OR 0.26 respectively; p < 0.05 in all cases) (Fig. 1; Fig. 2 centre panel; Suppl Table 2).

#### 3.1.3 Successful establishment of *S. pneumoniae* colonisation rather than challenge alone is associated with increased symptoms following TIV vaccination

**Fig. 3:**
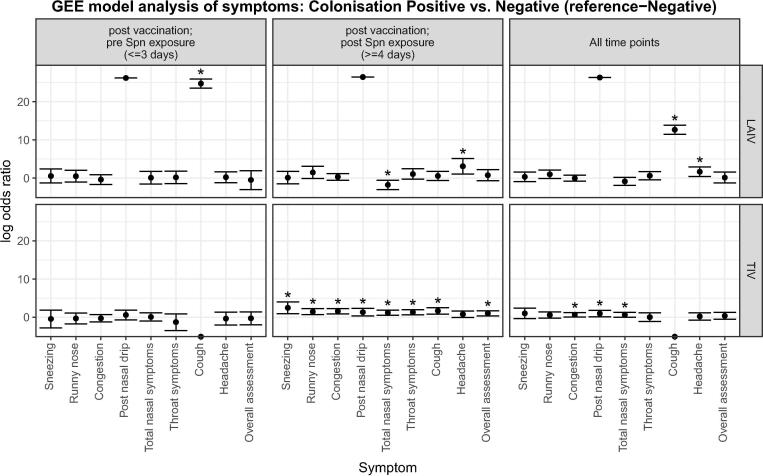
Antecedent Study- Symptoms reported by participants who had become colonised with *Streptococcus pneumoniae* (LAIV n = 24; TIV n = 23) compared with those who did not (LAIV n = 29; TIV n = 38). Timepoints are split up as until day of Spn inoculation, post Spn inoculation or all timepoints combined. Bars represent log_10_ transformed odds ratios with 95% confidence intervals. Asterisks denote p < 0.05 i.e. a statistically significant difference in the rate of reporting between colonised (positive) and non-colonised (negative) individuals. Odds ratios are derived from GEE model analysis (see methods), and are presented as those occurring early (left panels, at or before 3 days after vaccination and pre Spn inoculation), or late (centre panels, 4 or more days after vaccination and following Spn inoculation), or combined throughout the period of reporting (right panels). Reported symptoms are subdivided by their vaccination group: LAIV nasal spray (upper panels); TIV intramuscular (lower panels). Spn = *S. pneumoniae.*

**Fig. 4:**
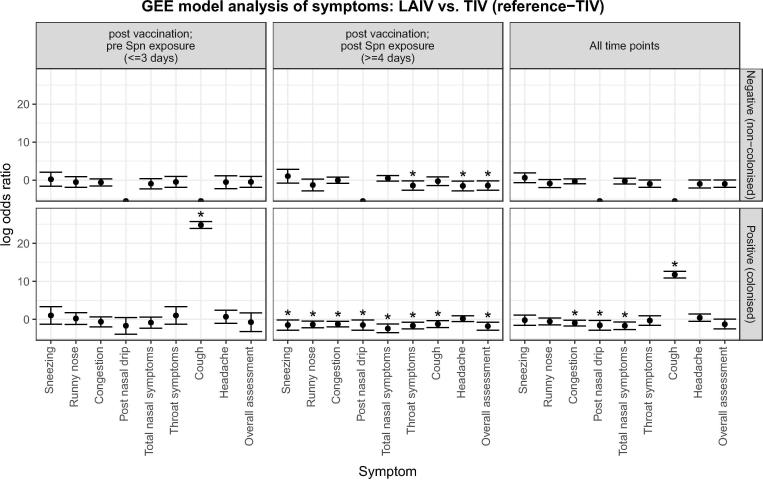
Antecedent study- Symptoms reported by participants receiving nasal LAIV (colonised/positive n = 24; non colonised/negative n = 29) compared with those receiving intramuscular TIV (colonised/positive n = 23; non colonised/negative n = 38). Timepoints are split up as until day of Spn inoculation, post Spn inoculation or all timepoints combined. Bars represent log_10_ transformed odds ratios with 95% confidence intervals. Asterisks denote p < 0.05 i.e. a statistically significant difference in the rate of reporting between the two arms of the trial. Odds ratios are derived from GEE model analysis (see methods), and are presented as those occurring early (left panels, at or before 3 days after vaccination and pre Spn inoculation), or late (centre panels, 4 or more days after vaccination and following Spn inoculation), or combined throughout the period of reporting (right panels). Reported symptoms are subdivided by their colonisation status: negative (no colonisation, upper panels); positive (colonised, lower panels). Spn = *S. pneumoniae.*

We investigated whether colonisation status led to symptoms following vaccination. In the TIV group, more nasal and non-nasal symptoms were reported by colonised participants (52%; n = 12/23) compared to non-colonised participants (34%; n = 13/38) following inoculation (>=4 days) with statistical significance seen for all reported nasal symptoms, sore throat, cough and overall assessment (Fig. 1; Fig. 3 lower centre panel; Suppl Table 3). In contrast, in the LAIV group, statistical difference was seen for only 2 symptoms. These were total nasal symptoms which were reduced and headache which was increased in colonised participants after inoculation (OR 0.17 and OR 22.17; p < 0.005) (Fig. 1; Fig. 3 upper centre panel; Suppl Table 4). Fewer nasal and non-nasal symptoms were reported by colonised participants who received LAIV prior to inoculation (38%; n = 9/24) compared to those participants who received TIV (52%; n = 12/23) (Fig. 1; Fig. 4 lower centre panel; Suppl Table 5). This statistical difference was observed mainly in nasal symptom reporting and for sore throat, cough and overall assessment (p < 0.05). Similarly, fewer symptoms were observed in non-colonised participants who received LAIV prior to inoculation (31%; n = 9/29) compared to those participants who received TIV (47%; n = 18/38). However, statistical significance was only seen in non-nasal symptoms; sore throat, headache and overall assessment (p < 0.05) (Fig. 1; Fig. 4 upper centre panel; Suppl Table 6).

In summary, similar rates of symptoms were reported following vaccination with either LAIV or TIV vaccinations. However, once participants were inoculated with *S. pneumoniae* statistical differences were seen in the reporting of symptoms between these two groups. Most noticeable was the reduction in symptoms reported in the LAIV group, regardless of colonisation status, compared to the TIV group.

### 3.2 Concurrent study

#### 3.2.1 LAIV and TIV do not differentially induce symptoms when administered after *S. pneumoniae*

**Fig. 5:**
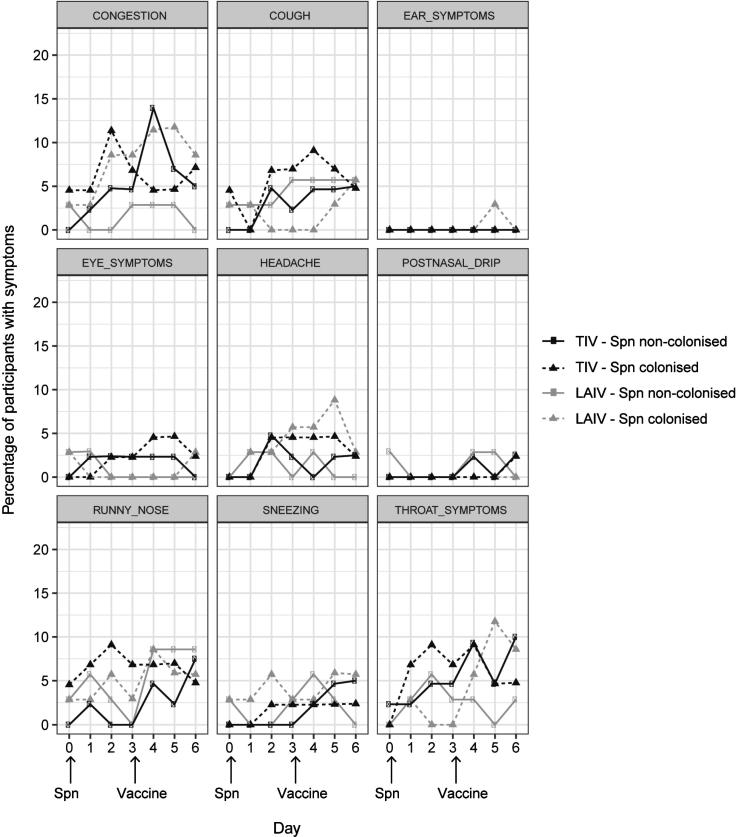
Symptom reporting in the concurrent cohort. The percentage of participants reporting symptoms per day for each of the collected symptoms per group: TIV – Spn non-colonised (n = 43, black circles and solid line), TIV – Spn colonised (n = 44, black triangles and dashed line), LAIV – Spn non-colonised (n = 35, grey circles and solid line), LAIV – Spn colonised (n = 35, grey triangles and dashed line). Day of vaccination and Spn inoculation are depicted. Spn = *S. pneumoniae.*

Participants were first inoculated with *S. pneumoniae* and then received influenza vaccines 3 days later. 163 participants completed the primary study end points and 157 returned their symptom log questionnaires for data analysis (LAIV n = 70, TIV n = 87). As reported, similar demographics were observed in both groups [18]. In total, 79 participants (50%) became experimentally colonised with both groups having similar colonisation rates (LAIV 50% and TIV 51%). Overall 31% (22/70) participants in the LAIV group and 32% (28/87) participants in the TIV group reported one or more nasal or non-nasal symptoms, or both, at any time point in the 6 days following inoculation. Runny nose (LAIV 11%, n = 8/70; TIV 13%, n = 11/87), congestion (LAIV 9%, n = 6/70; TIV 18%, n = 16/87) and sore throat (LAIV 11%, n = 8/70; TIV 14%, n = 12/87) were the most frequently reported symptoms in both groups. No statistical difference was seen in the reporting of symptoms across both groups (Fig. 5; Suppl Table 7).

We investigated whether inoculation with *S. pneumoniae* led to symptoms in healthy adults in the absence of any influenza vaccine in the three days prior to the administration of LAIV or TIV. At baseline (prior to inoculation) 10% (n = 7/10) in the LAIV group and 3% (n = 3/87) in the TIV group reported one or more nasal or non-nasal symptoms, or both. In the 3 days following inoculation similar rates of reported symptoms were noted across both groups (LAIV 21%, n = 15/70, TIV 22%, n = 19/87). Despite differences at baseline there was no statistical difference in the reporting of symptoms between the two groups (Fig. 5; Suppl Table 7). Following vaccination, three days after inoculation, symptoms remained at similar rates in both the LAIV group with 26% (n = 18/70) and 25% (n = 22/87) in the TIV group; no statistical difference was seen in the number of participants reporting symptoms following vaccination in either group (Fig. 5; Suppl Table 7).

#### 3.2.2 Nasal and non-nasal symptoms were the same between the LAIV and TIV group regardless of colonisation status

We investigated whether colonisation status led to symptoms following inoculation. There was no statistical difference in the symptoms reported by colonised participants by GEE (LAIV 37%, n = 13/35; TIV 39%, n = 17/44) compared to non-colonised participants (LAIV 26%, n = 9/35; TIV 26%, n = 11/43) following inoculation in either group (Fig. 5; suppl tables 8 and 9). Similarly, there was no statistical difference seen in the symptoms reported by colonised participants between the LAIV and TIV groups (Fig. 5; Suppl Table 10).

In summary, similar rates of symptom reporting was found in both groups following inoculation with *S. pneumoniae* and subsequently in the 3 days after receiving an influenza vaccination. Colonisation status of the participant did not affect the reporting of symptoms.

## 4 Discussion

This is the first study to evaluate symptoms associated with live attentuated influenza vaccine and controlled pneumococcal colonisation. The timing and route of influenza vaccination, and the presence of *S. pneumoniae* in the nasopharynx, had a significant effect on symptoms in adults. Reported symptoms following *S. pneumoniae* inoculation were consistent with those reported in previous EHPC studies [11]. However, higher incidences of nasal and non-nasal symptoms were reported by Trimble et al. [11] due to a lower threshold of symptom reporting. The most commonly reported symptoms following vaccination were runny nose, congestion, and sore throat; reflecting published data following LAIV administration [7]. However, we found much lower incidences of nasal symptoms in our study compared to previously published rates in adults following LAIV administration (20% vs 44%, respectively) [7]. Reasons for these differences is unclear but might relate to the timeframe of symptom data collection, year to year variation in vaccine replication efficiency, the different data collection methods and thresholds for reporting symptoms.

Our results demonstrate that LAIV administration prior to pneumococcal inoculation significantly reduces nasal and non-nasal symptoms compared to TIV. Additionally, less symptoms were seen in the LAIV group compared to the TIV group after inoculation despite similar symptom rates between the two groups following vaccination. For the concurrent study, there was no difference seen in symptom rates following inoculation or vaccination in either vaccine group. The reasons for these differences are unclear, although we have previously observed from the antecedent study that LAIV induces a strong pro-inflammatory response in the nose, but impairs nasal neutrophil responses to *S. pneumoniae* colonisation compared to TIV vaccinated participants [20]. During acute respiratory viral infections neutrophils have been shown to positively correlate with nasal symptom severity [28] and could explain why fewer nasal symptoms are reported in the LAIV vaccination group.

We found evidence that colonisation status led to significant differences in symptoms between the LAIV and TIV groups across both studies. In the antecedent study, TIV treated colonised adults had higher nasal symptom reporting compared to TIV non-colonised adults. However, in the LAIV group there was no difference seen in symptoms reported between colonised and non-colonised adults either before or after inoculation. Likewise, fewer nasal symptoms were seen in LAIV colonised adults compared to TIV colonised adults. In the concurrent study, colonisation status did not affect symptoms between the two vaccination groups. This suggests that TIV potentiates symptoms following colonisation. In the antecedent study, post nasal drip and cough were only increased pre but not post *S. pneumoniae* exposure. One possibility is that these are transient effects of vaccination, which can be observed in the first days following vaccine administration, but not at later points.

Co-colonisation (with LAIV and *S. pneumoniae*) in healthy adults led to a transient increase in acquisition at Day 2 following bacterial challenge in those who had previously received LAIV (33/55 [60.0%] vs 25/62 [40.3%] in TIV, p = 0.03). Bacterial carriage densities were increased approximately 10-fold by Day 9 in the LAIV recipients [18]. We have now shown that co-infection and its associated increase in pneumococcal density did not correlate with symptom reporting, consistent with other research in adults [11]. Conversely, in children, it has been found that being colonised with *S. pneumoniae* at the time of LAIV administration causes a 6-fold increase in bacterial densities at Day 28, with symptoms of rhinitis positively associated with pneumococcal density [17].

We used an adapted validated respiratory symptom tool [26], [29]. Recall bias was limited by contemporaneous completion of a symptom log. Using this novel human co-infection challenge model, we were able to determine the exact day of pneumococcal exposure in relation to the influenza vaccination and the timing and duration of pneumococcal colonisation. The main limitations were the use of two slightly different questionnaires between the antecedent and concurrent studies: we were therefore able to compare directly the presence of symptoms, but not severity. Secondly, there was no control group for mock-vaccinations or inoculations as it was assumed that in the absence of a vaccination or being non-colonised was an asymptomatic process [11], and we did not anticipate topical effects of TIV administration. A control group for mock-vaccinations or inoculations would have helped to further dissect the relative contributions of the vaccination and inoculation to symptoms.

We hypothesised that nasally administered LAIV would produce more symptoms than intramuscular TIV in healthy adults. However, the results, unexpectedly, demonstrated that TIV in particular when followed by *S. pneumoniae* colonisation caused more symptoms in adults than LAIV. These results were biologically relevant as we scored symptoms on a scale, where a score of ≤2 (no symptoms or occasional limited episode) was considered ‘asymptomatic’ and a score of ≥3 (mild steady symptoms to severe hard to tolerate symptoms) was considered ‘symptomatic’. By definition what we defined as symptomatic was therefore noticeable at least by the participants.

The authors acknowledge that these results are counter-intuitive based on the adverse events data collected following vaccination with LAIV versus TIV, previously [30]. However, we performed two randomised double-blinded placebo-controlled clinical trials with 271 participants who completed symptom questionnaires allowing us to interrogate the effects of vaccination in the setting of existing or new *S. pneumoniae* colonisation. The differences between groups were not apparent prior to the intervention, indicating these are true biological effects. What is important to note is that symptoms were driven not just by the vaccine alone, but strongly associated with the *S. pneuomoniae* colonisation afterwards. We demonstrated that LAIV impaired neutrophilic degranulation upon *S. pneumoniae* colonisation [20], and it is for example possible that this, or another immune mechanism that was altered by LAIV, led to the differences in symptoms. We believe these results highlight that the environment and microbiota can therefore affect symptomology differentially following vaccination with attenuated and inactivated vaccines. Importantly, the order of vaccination had an impact of symptom reporting, as it did on microbiological endpoints (*S. pneumoniae* colonisation) in the trial [18].

## 5 Conclusion

This is the first controlled challenge study in humans to directly assess the symptomology of a respiratory live viral vaccine during co-infection with a human pathogen. Our study demonstrates that symptoms experienced during live viral vaccination and bacterial co-infection in the nasopharynx are directly affected by the precedence of the pathogen acquisition. The mechanism by which LAIV and TIV influence the symptoms in adults remains undetermined. Further research should target our understanding of the immunological processes that underpin the symptomology of LAIV and TIV administration prior to or following the acquisition of *S. pneumoniae* in adults.

## Funding and sponsorship

The study was funded by the Bill and Melinda Gates Foundation (grant OPP1117728) and the UK Medical Research Council (grant MR/M011569/1). The study was co-sponsored by the Liverpool School of Tropical Medicine and the Royal Liverpool and Broadgreen University Hospitals NHS Trust, UK.

## CRediT authorship contribution statement

**Caz Hales:** Data curation, Formal analysis, Investigation, Project administration, Writing - original draft, Writing - review & editing. **Simon P. Jochems:** Data curation, Formal analysis, Project administration, Writing - original draft, Writing - review & editing. **Rachel Robinson:** Investigation, Writing - review & editing. **Carla Solórzano:** Writing - review & editing. **Beatriz Carniel:** Writing - review & editing. **Sherin Pojar:** Writing - review & editing. **Jesús Reiné:** Writing - review & editing. **Esther L. German:** Writing - review & editing. **Elissavet Nikolaou:** Writing - review & editing. **Elena Mitsi:** Writing - review & editing. **Angela D. Hyder-Wright:** Conceptualization, Funding acquisition, Investigation, Methodology, Project administration, Writing - review & editing. **Helen Hill:** Investigation, Project administration, Writing - review & editing. **Hugh Adler:** Investigation, Writing - review & editing. **Victoria Connor:** Investigation, Writing - review & editing. **Seher Zaidi:** Investigation, Writing - review & editing. **Catherine Lowe:** Investigation, Writing - review & editing. **Xiaojing Fan:** Formal analysis, Writing - review & editing. **Duolao Wang:** Formal analysis, Writing - review & editing. **Stephen B. Gordon:** Conceptualization, Funding acquisition, Methodology, Writing - review & editing. **Jamie Rylance:** Conceptualization, Data curation, Formal analysis, Funding acquisition, Methodology, Project administration, Writing - original draft, Writing - review & editing. **Daniela M. Ferreira:** Conceptualization, Data curation, Formal analysis, Funding acquisition, Methodology, Project administration, Writing - original draft, Writing - review & editing.

## Declaration of Competing Interest

The authors declare that they have no known competing financial interests or personal relationships that could have appeared to influence the work reported in this paper.
