# Supplementary material for: Symptoms associated with influenza vaccination and experimental human pneumococcal colonisation of the nasopharynx
Source: Vaccine. 2020 Feb 28;38(10):2298–306. doi: 10.1016/j.vaccine.2020.01.070 (PMC7045083; doi:10.1016/j.vaccine.2020.01.070)
Supplement: Supplementary data 2 [file mmc2.docx]

**Supplementary Tables**

**Supplementary Table 1:** Triggered clinical examinations of reported symptoms requiring clinical investigation at any time point in the study.

|  | Vaccine and Colonisation Status | Day  symptoms reported | Symptoms reported | Management plan /outcomes |
| --- | --- | --- | --- | --- |
| Antecedent study | | | | |
| 1 | LAIV positive | D9 | Sore throat, headache, cough | Viral infection (Respiratory syncytial virus PCR, conservative treatment) |
| 2 | TIV negative | D13 | Not recorded | Viral infection (GP visit, treated with aciclovir) |
| 3 | TIV negative | D24 | Flu-like symptoms, temp 38.8 | Non-significant illness (GP visit, conservative treatment) |
| 4 | TIV positive | D29 | Productive cough, myalgia, sore throat, generally unwell. | Viral infection (Coronavirus PCR, conservative treatment) |
| 5 | LAIV positive | D1 | D1 temperature 37.5, severe headache, sore throat with generalised tonsillar swelling | Non-significant illness (Viral PCR negative, conservative treatment). |
| 6 | LAIV positive | D10 | Sore throat | Alternative diagnosis (Coronavirus PCR, conservative treatment) |
| 7 | TIV positive | D1 | Temperature after inoculation. Pyrexia (>38.3), myalgia, sore throat, dry cough. | Viral infection (Influenza B PCR, conservative treatment) |
| 8 | TIV positive | D7 | Cough, sore throat, mild pharyngeal erythema | Viral infection (Viral PCR negative, conservative treatment) |
| Concurrent Study | | | | |
| 1 | TIV positive | D2 | Cough with yellow sputum | Non-significant illness (Viral PCR negative, conservative treatment) |
| 2 | TIV positive | D13 | Earache | Otitis externa (GP visit, treated with steroid/antibiotic drops) |
| 3 | TIV negative | D29 | Sore throat, cough | Viral infection (Respiratory syncytial virus PCR, conservative treatment) |
| 4 | TIV negative | D27 | Sore eyes | Conjunctivitis (treated with antibiotic ointment) |
| 5 | TIV negative | D2 | Bilateral red and inflamed eyes | Conjunctivitis (treated with chloramphenicol ointment) |
| 6 | LAIV negative | D9 | Sore throat, whitish spots | Non-significant illness (Viral PCR negative, conservative treatment). |
| 7 | TIV positive | D2 | Cough with phlegm, stuffed nose, generalised body aches, headaches, tiredness | Non-significant illness. Vaccination delayed to D6 |
| 8 | LAIV negative | D15 | Sore ears | Otitis externa (treated with steroid/antibiotic drops) |
| 9 | TIV positive | D21 | Productive cough, yellow phlegm, sore throat | Non-significant illness (Sputum sample no growth, conservative treatment). |
| 10 | LAIV positive | D3 | Pleuritic pain | Non-significant illness (clinical exam, ECG, D-dimer normal) |

**Supplementary Table 2:** Antecedent Study *GEE model analysis of symptoms: LAIV vs. TIV (reference-TIV)*

|  | | | | **95% CI** | |  |
| --- | --- | --- | --- | --- | --- | --- |
| **Parameter** | **Comparison** | **Days** | **OR** | **Lower Limit** | **Upper Limit** | **Probability** |
| Sneezing | LAIV vs. TIV | <=3 | 1.69 | 0.44 | 6.47 | 0.4452 |
|  |  | >=4 | 0.60 | 0.22 | 1.62 | 0.3168 |
|  |  | All time points | 1.01 | 0.44 | 2.34 | 0.9824 |
| Runny nose | LAIV vs. TIV | <=3 | 0.85 | 0.32 | 2.31 | 0.7570 |
|  |  | >=4 | **0.33** | 0.15 | 0.70 | **0.0040** |
|  |  | All time points | **0.53** | 0.28 | 0.99 | **0.0472** |
| Congestion | LAIV vs. TIV | <=3 | 0.56 | 0.26 | 1.19 | 0.1331 |
|  |  | >=4 | **0.55** | 0.32 | 0.95 | **0.0317** |
|  |  | All time points | **0.56** | 0.35 | 0.89 | **0.0138** |
| Itchy nose | LAIV vs. TIV | <=3 | . | . | . | . |
|  |  | >=4 | . | . | . | . |
|  |  | All time points | . | . | . | . |
| Post nasal drip | LAIV vs. TIV | <=3 | **0.11** | 0.01 | 0.88 | **0.0375** |
|  |  | >=4 | **0.19** | 0.05 | 0.65 | **0.0080** |
|  |  | All time points | **0.14** | 0.04 | 0.48 | **0.0017** |
| Total nasal symptoms | LAIV vs. TIV | <=3 | 0.42 | 0.16 | 1.11 | 0.0805 |
|  |  | >=4 | **0.57** | 0.33 | 0.98 | **0.0430** |
|  |  | All time points | **0.49** | 0.28 | 0.86 | **0.0121** |
| Eye symptoms | LAIV vs. TIV | <=3 | . | . | . | . |
|  |  | >=4 | . | . | . | . |
|  |  | All time points | . | . | . | . |
| Throat symptoms | LAIV vs. TIV | <=3 | 1.02 | 0.34 | 3.12 | 0.9674 |
|  |  | >=4 | **0.26** | 0.13 | 0.53 | **0.0002** |
|  |  | All time points | 0.52 | 0.27 | 1.01 | 0.0522 |
| Cough | LAIV vs. TIV | <=3 | 3.31 | 0.63 | 17.38 | 0.1578 |
|  |  | >=4 | **0.47** | 0.24 | 0.94 | **0.0338** |
|  |  | All time points | 1.25 | 0.51 | 3.08 | 0.6243 |
| Ear symptoms | LAIV vs. TIV | <=3 | . | . | . | . |
|  |  | >=4 | . | . | . | . |
|  |  | All time points | . | . | . | . |
| Headache | LAIV vs. TIV | <=3 | 1.37 | 0.48 | 3.88 | 0.5538 |
|  |  | >=4 | 0.83 | 0.44 | 1.57 | 0.5654 |
|  |  | All time points | 1.07 | 0.58 | 1.97 | 0.8405 |
| Overall assessment | LAIV vs. TIV | <=3 | 0.54 | 0.14 | 2.14 | 0.3825 |
|  |  | >=4 | **0.22** | 0.10 | 0.47 | **0.0001** |
|  |  | All time points | **0.34** | 0.16 | 0.76 | **0.0081** |

**Bold** indicates statistically significant difference (p<0.05) in the odds of reporting between the two arms of the trial

Missing values indicate that there was no need to use the GEE model because there was not enough data values.

**Supplementary Table 3**: Antecedent Study GEE model analysis of symptoms-TIV: Colonisation Positive vs. Negative (reference-Negative)

|  | | | | **95% CI** | |  |
| --- | --- | --- | --- | --- | --- | --- |
| **Parameter** | **Comparison** | **Days** | **OR** | **Lower Limit** | **Upper Limit** | **Probability** |
| Sneezing | Positive vs. Negative | <=3 | 0.64 | 0.06 | 6.35 | 0.7034 |
|  |  | >=4 | **11.75** | 2.54 | 54.38 | **0.0016** |
|  |  | All time points | 2.74 | 0.69 | 10.92 | 0.1522 |
| Runny nose | Positive vs. Negative | <=3 | 0.73 | 0.18 | 2.96 | 0.6644 |
|  |  | >=4 | **4.31** | 1.97 | 9.45 | **0.0003** |
|  |  | All time points | 1.78 | 0.80 | 3.97 | 0.1585 |
| Congestion | Positive vs. Negative | <=3 | 0.76 | 0.29 | 1.96 | 0.5675 |
|  |  | >=4 | **4.80** | 2.44 | 9.43 | **<.0001** |
|  |  | All time points | **1.91** | 1.06 | 3.43 | **0.0309** |
| Itchy nose | Positive vs. Negative | <=3 | . | . | . | . |
|  |  | >=4 | . | . | . | . |
|  |  | All time points | . | . | . | . |
| Post nasal drip | Positive vs. Negative | <=3 | 1.81 | 0.50 | 6.55 | 0.3681 |
|  |  | >=4 | **3.72** | 1.35 | 10.22 | **0.0110** |
|  |  | All time points | **2.59** | 1.14 | 5.91 | **0.0235** |
| Total nasal symptoms | Positive vs. Negative | <=3 | 1.07 | 0.37 | 3.12 | 0.8955 |
|  |  | >=4 | **3.30** | 1.70 | 6.43 | **0.0004** |
|  |  | All time points | **1.88** | 1.00 | 3.54 | **0.0494** |
| Eye symptoms | Positive vs. Negative | <=3 | . | . | . | . |
|  |  | >=4 | . | . | . | . |
|  |  | All time points | . | . | . | . |
| Throat symptoms | Positive vs. Negative | <=3 | 0.28 | 0.03 | 2.41 | 0.2487 |
|  |  | >=4 | **3.66** | 1.86 | 7.19 | **0.0002** |
|  |  | All time points | 1.02 | 0.33 | 3.14 | 0.9752 |
| Cough | Positive vs. Negative | <=3 | . | . | . | . |
|  |  | >=4 | . | . | . | . |
|  |  | All time points | . | . | . | . |
| Ear symptoms | Positive vs. Negative | <=3 | . | . | . | . |
|  |  | >=4 | . | . | . | . |
|  |  | All time points | . | . | . | . |
| Headache | Positive vs. Negative | <=3 | 0.70 | 0.13 | 3.73 | 0.6737 |
|  |  | >=4 | 2.20 | 0.95 | 5.10 | 0.0657 |
|  |  | All time points | 1.24 | 0.48 | 3.18 | 0.6547 |
| Overall assessment | Positive vs. Negative | <=3 | 0.76 | 0.14 | 4.07 | 0.7483 |
|  |  | >=4 | **2.78** | 1.44 | 5.36 | **0.0023** |
|  |  | All time points | 1.45 | 0.59 | 3.59 | 0.4179 |

**Bold** indicates statistically significant difference (p<0.05) in the odds of reporting between colonised and non-colonised TIV participants.

Missing values indicate that there was no need to use the GEE model because there was not enough data values.

**Supplementary Table 4**: Antecedent Study GEE model analysis of symptoms-LAIV: Colonisation Positive vs. Negative (reference-Negative)

|  | | | | **95% CI** | |  |
| --- | --- | --- | --- | --- | --- | --- |
| **Parameter** | **Comparison** | **Days** | **OR** | **Lower Limit** | **Upper Limit** | **Probability** |
| Sneezing | Positive vs. Negative | <=3 | 1.73 | 0.28 | 10.82 | 0.5582 |
|  |  | >=4 | 1.13 | 0.22 | 5.73 | 0.8832 |
|  |  | All time points | 1.40 | 0.41 | 4.77 | 0.5934 |
| Runny nose | Positive vs. Negative | <=3 | 1.65 | 0.35 | 7.71 | 0.5269 |
|  |  | >=4 | 4.39 | 0.89 | 21.59 | 0.0684 |
|  |  | All time points | 2.69 | 0.89 | 8.16 | 0.0807 |
| Congestion | Positive vs. Negative | <=3 | 0.69 | 0.19 | 2.46 | 0.5627 |
|  |  | >=4 | 1.39 | 0.57 | 3.38 | 0.4670 |
|  |  | All time points | 0.98 | 0.45 | 2.13 | 0.9515 |
| Itchy nose | Positive vs. Negative | <=3 | 1.01 | 0.00 | . | 1.0000 |
|  |  | >=4 | 2.52 | 0.45 | 14.05 | 0.2932 |
|  |  | All time points | 1.59 | 0.00 | . | 1.0000 |
| Post nasal drip | Positive vs. Negative | <=3 | . | . | . | . |
|  |  | >=4 | . | . | . | . |
|  |  | All time points | . | . | . | . |
| Total nasal symptoms | Positive vs. Negative | <=3 | 1.09 | 0.21 | 5.64 | 0.9151 |
|  |  | >=4 | **0.17** | 0.05 | 0.58 | **0.0047** |
|  |  | All time points | 0.42 | 0.15 | 1.20 | 0.1049 |
| Throat symptoms | Positive vs. Negative | <=3 | 1.21 | 0.24 | 6.19 | 0.8219 |
|  |  | >=4 | 2.87 | 0.73 | 11.34 | 0.1318 |
|  |  | All time points | 1.86 | 0.64 | 5.43 | 0.2550 |
| Cough | Positive vs. Negative | <=3 | . | . | . | . |
|  |  | >=4 | . | . | . | . |
|  |  | All time points | . | . | . | . |
| Ear symptoms | Positive vs. Negative | <=3 | . | . | . | . |
|  |  | >=4 | . | . | . | . |
|  |  | All time points | . | . | . | . |
| Headache | Positive vs. Negative | <=3 | 1.27 | 0.30 | 5.28 | 0.7434 |
|  |  | >=4 | **22.17** | 2.90 | 169.53 | **0.0028** |
|  |  | All time points | 5.30 | 1.53 | 18.41 | 0.0086 |
| Overall assessment | Positive vs. Negative | <=3 | 0.62 | 0.05 | 7.03 | 0.7007 |
|  |  | >=4 | 2.13 | 0.50 | 9.10 | 0.3097 |
|  |  | All time points | 1.15 | 0.28 | 4.75 | 0.8477 |

**Bold** indicates statistically significant difference (p<0.05) in the odds of reporting between colonised and non-colonised LAIV participants

Missing values indicate that there was no need to use the GEE model because there was not enough data values.

**Supplementary Table 5**: Antecedent Study GEE model analysis of symptoms: LAIV vs. TIV only for Colonisation Positive (reference-TIV)

|  | | | | **95% CI** | |  |
| --- | --- | --- | --- | --- | --- | --- |
| **Parameter** | **Comparison** | **Days** | **OR** | **Lower Limit** | **Upper Limit** | **Probability** |
| Sneezing | LAIV vs. TIV | <=3 | 2.85 | 0.28 | 28.49 | 0.3729 |
|  |  | >=4 | **0.23** | 0.06 | 0.86 | **0.0290** |
|  |  | All time points | 0.81 | 0.21 | 3.07 | 0.7594 |
| Runny nose | LAIV vs. TIV | <=3 | 1.27 | 0.27 | 5.95 | 0.7609 |
|  |  | >=4 | **0.26** | 0.11 | 0.64 | **0.0034** |
|  |  | All time points | 0.58 | 0.24 | 1.42 | 0.2314 |
| Congestion | LAIV vs. TIV | <=3 | 0.53 | 0.14 | 1.92 | 0.3297 |
|  |  | >=4 | **0.29** | 0.14 | 0.60 | **0.0009** |
|  |  | All time points | 0.39 | 0.18 | 0.82 | 0.0129 |
| Itchy nose | LAIV vs. TIV | <=3 | 1.10 | 0.00 | . | 1.0000 |
|  |  | >=4 | 0.45 | 0.13 | 1.51 | 0.1968 |
|  |  | All time points | 0.70 | 0.00 | . | 1.0000 |
| Post nasal drip | LAIV vs. TIV | <=3 | 0.19 | 0.02 | 1.65 | 0.1307 |
|  |  | >=4 | **0.23** | 0.06 | 0.85 | **0.0269** |
|  |  | All time points | 0.21 | 0.06 | 0.74 | 0.0152 |
| Total nasal symptoms | LAIV vs. TIV | <=3 | 0.43 | 0.10 | 1.82 | 0.2520 |
|  |  | >=4 | **0.09** | 0.03 | 0.30 | **<.0001** |
|  |  | All time points | 0.19 | 0.07 | 0.50 | 0.0007 |
| Eye symptoms | LAIV vs. TIV | <=3 | 1.05 | 0.00 | . | 1.0000 |
|  |  | >=4 | 0.50 | 0.09 | 2.77 | 0.4260 |
|  |  | All time points | 0.72 | 0.00 | . | 1.0000 |
| Throat symptoms | LAIV vs. TIV | <=3 | 2.81 | 0.28 | 27.93 | 0.3789 |
|  |  | >=4 | **0.19** | 0.08 | 0.47 | **0.0003** |
|  |  | All time points | 0.74 | 0.21 | 2.53 | 0.6284 |
| Cough | LAIV vs. TIV | <=3 | . | . | . | . |
|  |  | >=4 | . | . | . | . |
|  |  | All time points | . | . | . | . |
| Ear symptoms | LAIV vs. TIV | <=3 | 1.03 | 0.00 | . | 1.0000 |
|  |  | >=4 | 0.65 | 0.18 | 2.36 | 0.5107 |
|  |  | All time points | 0.82 | 0.00 | . | 1.0000 |
| Headache | LAIV vs. TIV | <=3 | 2.01 | 0.35 | 11.35 | 0.4307 |
|  |  | >=4 | 1.22 | 0.57 | 2.63 | 0.6126 |
|  |  | All time points | 1.56 | 0.61 | 4.04 | 0.3550 |
| Overall assessment | LAIV vs. TIV | <=3 | 0.48 | 0.04 | 5.44 | 0.5514 |
|  |  | >=4 | **0.17** | 0.06 | 0.46 | **0.0005** |
|  |  | All time points | 0.28 | 0.08 | 1.06 | 0.0612 |

**Bold** indicates statistically significant difference (p<0.05) in the odds of reporting between the two arms of the trial

Missing values indicate that there was no need to use the GEE model because there was not enough data values.

**Supplementary Table 6**: Antecedent Study GEE model analysis of symptoms: LAIV vs. TIV only for Colonisation Negative (reference-TIV)

|  | | | | **95% CI** | |  |
| --- | --- | --- | --- | --- | --- | --- |
| **Parameter** | **Comparison** | **Days** | **OR** | **Lower Limit** | **Upper Limit** | **Probability** |
| Sneezing | LAIV vs. TIV | <=3 | 1.25 | 0.20 | 7.88 | 0.8125 |
|  |  | >=4 | 2.85 | 0.46 | 17.50 | 0.2583 |
|  |  | All time points | 1.89 | 0.52 | 6.91 | 0.3377 |
| Runny nose | LAIV vs. TIV | <=3 | 0.60 | 0.15 | 2.48 | 0.4839 |
|  |  | >=4 | 0.28 | 0.06 | 1.32 | 0.1081 |
|  |  | All time points | 0.41 | 0.14 | 1.18 | 0.0996 |
| Congestion | LAIV vs. TIV | <=3 | 0.55 | 0.21 | 1.43 | 0.2219 |
|  |  | >=4 | 0.97 | 0.42 | 2.28 | 0.9530 |
|  |  | All time points | 0.73 | 0.38 | 1.40 | 0.3457 |
| Itchy nose | LAIV vs. TIV | <=3 | . | . | . | . |
|  |  | >=4 | . | . | . | . |
|  |  | All time points | . | . | . | . |
| Post nasal drip | LAIV vs. TIV | <=3 | . | . | . | . |
|  |  | >=4 | . | . | . | . |
|  |  | All time points | . | . | . | . |
| Total nasal symptoms | LAIV vs. TIV | <=3 | 0.39 | 0.10 | 1.48 | 0.1672 |
|  |  | >=4 | 1.61 | 0.79 | 3.28 | 0.1894 |
|  |  | All time points | 0.79 | 0.37 | 1.70 | 0.5526 |
| Eye symptoms | LAIV vs. TIV | <=3 | . | . | . | . |
|  |  | >=4 | . | . | . | . |
|  |  | All time points | . | . | . | . |
| Throat symptoms | LAIV vs. TIV | <=3 | 0.62 | 0.15 | 2.64 | 0.5178 |
|  |  | >=4 | **0.24** | 0.07 | 0.85 | **0.0274** |
|  |  | All time points | 0.38 | 0.15 | 1.02 | 0.0538 |
| Cough | LAIV vs. TIV | <=3 | . | . | . | . |
|  |  | >=4 | . | . | . | . |
|  |  | All time points | . | . | . | . |
| Ear symptoms | LAIV vs. TIV | <=3 | 1.02 | 0.26 | 4.01 | 0.9773 |
|  |  | >=4 | 0.11 | 0.01 | 0.87 | 0.0365 |
|  |  | All time points | 0.34 | 0.10 | 1.17 | 0.0860 |
| Headache | LAIV vs. TIV | <=3 | 0.60 | 0.11 | 3.18 | 0.5441 |
|  |  | >=4 | **0.22** | 0.06 | 0.78 | **0.0186** |
|  |  | All time points | 0.37 | 0.13 | 1.04 | 0.0601 |
| Overall assessment | LAIV vs. TIV | <=3 | 0.62 | 0.15 | 2.64 | 0.5178 |
|  |  | **>=4** | 0.24 | 0.07 | 0.85 | **0.0274** |
|  |  | All time points | 0.38 | 0.15 | 1.02 | 0.0538 |

**Bold** indicates statistically significant difference (p<0.05) in the odds of reporting between the two arms of the trial

Missing values indicate that there was no need to use the GEE model because there was not enough data values.

**Supplementary Table 7**: Concurrent Study GEE model analysis of symptoms: LAIV VS TIV (reference-TIV)

|  | | | | ***95% CI*** | |  |
| --- | --- | --- | --- | --- | --- | --- |
| **Parameter** | **Comparison** | **Day** | **OR** | **Lower Limit** | **Upper Limit** | **Probability** |
| SNEEZING | LAIV VS TIV | <=3 | . | . | . | . |
|  |  | >=4 | . | . | . | . |
|  |  | All time points | . | . | . | . |
| RUNNY NOSE | LAIV VS TIV | <=3 | 0.86 | 0.18 | 4.03 | 0.8481 |
|  |  | >=4 | 1.44 | 0.41 | 5.01 | 0.5683 |
|  |  | All time points | 1.11 | 0.41 | 3.00 | 0.8343 |
| CONGESTION | LAIV VS TIV | <=3 | 0.36 | 0.04 | 3.57 | 0.3809 |
|  |  | >=4 | 0.66 | 0.20 | 2.20 | 0.5017 |
|  |  | All time points | 0.49 | 0.13 | 1.78 | 0.2772 |
| POST NASAL DRIP | LAIV VS TIV | <=3 | . | . | . | . |
|  |  | >=4 | . | . | . | . |
|  |  | All time points | . | . | . | . |
| EYE SYMPTOMS | LAIV VS TIV | <=3 | . | . | . | . |
|  |  | >=4 | . | . | . | . |
|  |  | All time points | . | . | . | . |
| THROAT SYMPTOMS | LAIV VS TIV | <=3 | 0.61 | 0.11 | 3.45 | 0.5773 |
|  |  | >=4 | 0.44 | 0.11 | 1.74 | 0.2435 |
|  |  | All time points | 0.52 | 0.17 | 1.57 | 0.2465 |
| COUGH | LAIV VS TIV | <=3 | . | . | . | . |
|  |  | >=4 | . | . | . | . |
|  |  | All time points | . | . | . | . |
| HEADACHE | LAIV VS TIV | <=3 | . | . | . | . |
|  |  | >=4 | . | . | . | . |
|  |  | All time points | . | . | . | . |

**Bold** indicates statistically significant difference (p<0.05) in the odds of reporting between the two arms of the trial

Missing values indicate that there was no need to use the GEE model because there was not enough data values.

**Supplementary Table 8**: Concurrent study GEE model analysis of symptoms-LAIV Colonisation Positive vs. Negative (reference-Negative)

|  | | | | **95% CI** | |  |
| --- | --- | --- | --- | --- | --- | --- |
| **Parameter** | **Comparison** | **Day** | **OR** | **Lower Limit** | **Upper Limit** | **Probability** |
| SNEEZING | Positive VS Negative | <=3 | . | . | . | . |
|  |  | >=4 | . | . | . | . |
|  |  | All time points | . | . | . | . |
| RUNNYNOSE | Positive VS Negative | <=3 | 0.54 | 0.05 | 6.37 | 0.6231 |
|  |  | >=4 | 1.13 | 0.20 | 6.22 | 0.8910 |
|  |  | All time points | 0.78 | 0.17 | 3.51 | 0.7449 |
| CONGESTION | Positive VS Negative | <=3 | . | . | . | . |
|  |  | >=4 | . | . | . | . |
|  |  | All time points | . | . | . | . |
| THROATSYMPTOMS | Positive VS Negative | <=3 | 1.15 | 0.07 | 19.58 | 0.9221 |
|  |  | >=4 | 2.42 | 0.20 | 28.66 | 0.4837 |
|  |  | All time points | 1.67 | 0.25 | 10.94 | 0.5933 |
| COUGH | Positive VS Negative | <=3 | . | . | . | . |
|  |  | >=4 | . | . | . | . |
|  |  | All time points | . | . | . | . |
| HEADACHE | Positive VS Negative | <=3 | 1.25 | 0.07 | 22.13 | 0.8790 |
|  |  | >=4 | 2.73 | 0.22 | 34.01 | 0.4358 |
|  |  | All time points | 1.85 | 0.27 | 12.50 | 0.5297 |

**Bold** indicates statistically significant difference (p<0.05) in the odds of reporting between colonised and non-colonised LAIV participants

Missing values indicate that there was no need to use the GEE model because there was not enough data values.

**Supplementary Table 9**: Concurrent study GEE model analysis of symptoms-TIV Colonisation Positive vs. Negative (reference-Negative)

|  | | | | **95% CI** | |  |
| --- | --- | --- | --- | --- | --- | --- |
| **Parameter** | **Comparison** | **Day** | **OR** | **Lower Limit** | **Upper Limit** | **Probability** |
| SNEEZING | Positive VS Negative | <=3 | 1.06 | 0.00 | . | 1.0000 |
|  |  | >=4 | 1.03 | 0.06 | 17.53 | 0.9827 |
|  |  | All time points | 1.04 | 0.00 | . | 1.0000 |
| RUNNYNOSE | Positive VS Negative | <=3 | 2.83 | 0.28 | 28.86 | 0.3793 |
|  |  | >=4 | 1.37 | 0.21 | 8.85 | 0.7394 |
|  |  | All time points | 1.97 | 0.44 | 8.78 | 0.3729 |
| CONGESTION | Positive VS Negative | <=3 | 1.64 | 0.14 | 19.46 | 0.6945 |
|  |  | >=4 | 0.21 | 0.04 | 1.20 | 0.0796 |
|  |  | All time points | 0.59 | 0.13 | 2.68 | 0.4957 |
| POSTNASALDRIP | Positive VS Negative | <=3 | 0.82 | 0.05 | 14.06 | 0.8915 |
|  |  | >=4 | 0.82 | 0.05 | 14.06 | 0.8915 |
|  |  | All time points | 0.82 | 0.11 | 6.16 | 0.8476 |
| EYESYMPTOMS | Positive VS Negative | <=3 | 0.00 | 0.00 | . | 0.9999 |
|  |  | >=4 | 1.71 | 0.14 | 20.39 | 0.6722 |
|  |  | All time points | 0.00 | 0.00 | . | 0.9999 |
| THROATSYMPTOMS | Positive VS Negative | <=3 | 2.83 | 0.28 | 28.69 | 0.3781 |
|  |  | >=4 | 0.89 | 0.20 | 3.88 | 0.8743 |
|  |  | All time points | 1.59 | 0.40 | 6.28 | 0.5114 |
| COUGH | Positive VS Negative | <=3 | 0.92 | 0.00 | . | 1.0000 |
|  |  | >=4 | 1.90 | 0.32 | 11.18 | 0.4792 |
|  |  | All time points | 1.32 | 0.00 | . | 1.0000 |

**Bold** indicates statistically significant difference (p<0.05) in the odds of reporting between colonised and non-colonised TIV participants

Missing values indicate that there was no need to use the GEE model because there was not enough data values.

**Supplementary Table 10**: Concurrent study GEE model analysis of symptoms: LAIV vs. TIV only for Colonisation Positive (reference-TIV)

|  | | | | **95% CI** | |  |
| --- | --- | --- | --- | --- | --- | --- |
| **Parameter** | **Comparison** | **Day** | **OR** | **Lower Limit** | **Upper Limit** | **Probability** |
| SNEEZING | LAIV VS TIV | <=3 | . | . | . | . |
|  |  | >=4 | . | . | . | . |
|  |  | All time points | . | . | . | . |
| RUNNYNOSE | LAIV VS TIV | <=3 | 0.41 | 0.04 | 4.28 | 0.4557 |
|  |  | >=4 | 1.38 | 0.25 | 7.72 | 0.7162 |
|  |  | All time points | 0.75 | 0.18 | 3.22 | 0.6996 |
| CONGESTION | LAIV VS TIV | <=3 | 0.64 | 0.05 | 7.62 | 0.7241 |
|  |  | >=4 | 3.04 | 0.49 | 18.66 | 0.2305 |
|  |  | All time points | 1.39 | 0.30 | 6.48 | 0.6716 |
| THROATSYMPTOMS | LAIV VS TIV | <=3 | 0.44 | 0.04 | 4.46 | 0.4873 |
|  |  | >=4 | 0.67 | 0.11 | 3.90 | 0.6518 |
|  |  | All time points | 0.54 | 0.13 | 2.32 | 0.4090 |
| COUGH | LAIV VS TIV | <=3 | . | . | . | . |
|  |  | >=4 | 0.00 | 0.00 | 0.00 | <.0001 |
|  |  | All time points | 0.52 | 0.05 | 4.98 | 0.5707 |
| HEADACHE | LAIV VS TIV | <=3 | . | . | . | . |
|  |  | >=4 | . | . | . | . |
|  |  | All time points | . | . | . | . |

**Bold** indicates statistically significant difference (p<0.05) in the odds of reporting between the two arms of the trial

Missing values indicate that there was no need to use the GEE model because there was not enough data values.
